# General practitioners’ perspectives on campaigns to promote rapid help-seeking behaviour at the onset of rheumatoid arthritis

**DOI:** 10.3109/02813432.2014.900239

**Published:** 2014-03

**Authors:** Rebecca J. Stack, Zara Llewellyn, Chris Deighton, Patrick Kiely, Christian D. Mallen, Karim Raza

**Affiliations:** ^1^Sandwell and West Birmingham Hospitals NHS Trust, Birmingham, UK; ^2^Centre for Translational Inflammation Research, University of Birmingham, Birmingham, UK; ^3^Department of Rheumatology, Derby Hospitals NHS Foundation Trust, Derby, UK; ^4^Department of Rheumatology, St Georges Healthcare Trust, London, UK; ^5^Arthritis Research UK Primary Care Centre, University of Keele, UK

**Keywords:** General practice, general practitioner, health promotion, primary care, qualitative, rheumatoid arthritis, seeking help, United Kingdom

## Abstract

*Objective*. To explore general practitioners’ (GPs’) perspectives on public health campaigns to encourage people with the early symptoms of rheumatoid arthritis (RA) to seek medical help rapidly. *Design*. Nineteen GPs participated in four semi-structured focus groups. Focus groups were audio-recorded, transcribed verbatim, and analysed using thematic analysis. *Results*. GPs recognised the need for the early treatment of RA and identified that facilitating appropriate access to care was important. However, not all held the view that a delay in help seeking was a clinically significant issue. Furthermore, many were concerned that the early symptoms of RA were often non-specific, and that current knowledge about the nature of symptoms at disease onset was inadequate to inform the content of a help-seeking campaign. They argued that a campaign might not be able to specifically target those who need to present urgently. Poorly designed campaigns were suggested to have a negative impact on GPs’ workloads, and would “clog up” the referral pathway for genuine cases of RA. *Conclusions*. GPs were supportive of strategies to improve access to Rheumatological care and increase public awareness of RA symptoms. However, they have identified important issues that need to be considered in developing a public health campaign that forms part of an overall strategy to reduce time to treatment for patients with new onset RA. This study highlights the value of gaining GPs’ perspectives before launching health promotion campaigns.

- Campaigns to encourage prompt help-seeking behaviour at rheumatoid arthritis (RA) onset are vital to ensure early treatment.
- This study explored general practitioners’ (GPs’) perspectives on the impact of public health campaigns on primary care services.
- GPs were concerned that poorly constructed campaigns unduly worried the public and pressurised primary care services and referral pathways.
- GPs described poor understandings of early RA symptoms, highlighting the need to strengthen the evidence base in this respect to inform campaigns.

## Background

The early treatment of patients with rheumatoid arthritis (RA) is essential. The first three months following RA onset represent a therapeutic window during which disease-modifying anti-rheumatic drug (DMARD) treatment is particularly effective at controlling synovitis and limiting subsequent joint damage (1–6). However, there are considerable delays between symptom onset and the initiation of therapy (7–14). Delays can occur at multiple points in the patient's journey including patient delays in seeking medical advice at symptom onset, delays by general practitioners (GPs) in recognizing symptoms and referring the patient to a rheumatologist, and delays in secondary care (15–17). Recent studies from across Europe have found that many patients missed the potential therapeutic window because they delayed seeking help for their symptoms (8,18). A meta-synthesis identified that early symptom experiences, speaking to other people, and gathering information about symptoms were important drivers of help-seeking behaviour at RA onset (19). The review also identified that people had little knowledge of RA before diagnosis, believing RA to be a mild condition that affected older people. These misperceptions made correct symptom interpretation unlikely, highlighting the need for interventions targeted at understandings of RA help-seeking behaviours.

A report has highlighted the need for strategies to reduce the time to initiation of DMARDs in RA patients, with the promotion of help seeking being a key aspect (20). In response to concerns that the public needs more information about RA and its symptoms, (16,20,21) organisations such as the American College of Rheumatology and the UK's National Rheumatoid Arthritis Society have launched campaigns targeted at the public (22,23). Interventions to encourage people to seek help rapidly for inflammatory joint symptoms will have an impact on primary care workloads. For example, a Cochrane review of public health campaigns showed consistently increased service utilisation following exposure (24). Therefore, before interventions to increase rapid help-seeking behaviour are widely implemented, it is important to ascertain the views of GPs and to incorporate these into relevant public health strategies.

## Material and methods

The aim of this study was to explore GPs’ views regarding the potential advantages, disadvantages, and challenges associated with a large-scale public health intervention highlighting the symptoms of RA and encouraging rapid help seeking.

This research was approved by South Birmingham Ethics committee (10/H1207/98) and all participants gave written informed consent.

### Participants

GPs were identified and approached through a local primary care email distribution list. Invitations to participate were sent to 30 GPs. A self-selecting sample of 19 GPs participated, of whom eight were female. Participants served a range of rural and urban practices. The experience of GPs ranged from recent qualification to 28 years of primary care experience.

### Procedure

Four focus groups led by a facilitator (ZL or RJS) were undertaken. The discussions, guided by a schedule, explored GPs’ perspectives on the feasibility and acceptability of strategies to promote help-seeking behaviour in RA, their knowledge of current help-seeking campaigns, and their views about the drivers for, and barriers to, patient help seeking at the onset of RA. The facilitators used probing questions and prompts where appropriate to allow in-depth discussions of the questions set in the schedule. The focus-group topic schedule was piloted with a GP in a face-to-face interview (not used as part of the analysis described in the findings). Following this pilot interview, the schedule was revised (Table I).

**Table I.** Focus-group topic schedule.

The management of early RA and seeking help at the onset of RA:
• What are the symptoms you see as being indicative of a person with new-onset RA?
• What are your thoughts about the urgency of treatment in patients with a new onset of RA?
• What are your thoughts about the ease of diagnosis of RA?
• Do you think there are any issues about how quickly you are able to see patients with a new onset of RA (from the time their symptoms first began)?
• When patients present with a new-onset RA what, in your opinion, are their reasons for presentation?
• How aware/unaware do you think your patients are about RA?
• What do you believe are the reasons that people delay in their presentation with RA?
•How well educated do you believe allied healthcare professionals (physiotherapists, pharmacists) are around inflammatory arthritides?
• How do you believe the interaction between primary and secondary care affects the diagnosis and treatment of patients with RA?
• Safety-netting is common practice in general practice; how does this influence your treatment of patients with potential RA?
• Interventions and campaigns:
• Do you think we need approaches to get patients to see you sooner after the onset of their RA?
• Are you aware of any health campaigns surrounding RA? What do you think the strengths/weaknesses of this campaign are?
• What in general are your thoughts about a public health campaign encouraging patients to present early with symptoms of RA?
• What do you think the messages should be?
• What would be the problems/threats/benefits associated with such a campaign?
• From your perspective, how could a campaign be constructed to minimise problems and maximise benefits?
• Public health campaigns are not always welcomed favourably by GPs. What form of public education is the most successful?
• Closing question
• Is there anything else, relating to encouraging patients with a new onset of RA to seek help earlier, that we've not covered and that you want to tell me about?

Each focus group lasted approximately one hour. The discussions were audio-recorded and transcribed verbatim. Data were collected until thematic saturation had been reached and no new themes were emerging from the data collected. Data collection, transcribing, and analysis of interviews were undertaken in parallel allowing themes derived from earlier interviews to inform later interviews.

Data were analysed using thematic analysis (25) and this was facilitated by NVIVO (26). Initial coding was used to generate analytical summaries, which were grouped together into the most noteworthy and frequently occurring categories. Validation of initial coding was undertaken on one of the focus-group transcripts (by ZL and RJS). Blind coding was used to develop reliable and inclusive themes. Coding categories that lacked concordance were discussed and absorbed into the coding framework.

## Results

The analysis generated three themes relevant to GPs’ perspectives on campaigns to promote help seeking in people with the symptoms of new-onset RA (results are summarised in Table II). The themes identified were the need for early intervention and for a campaign, concerns about a future help-seeking campaign for RA, and GPs’ perspectives on the features of “good” and “bad” campaigns.

**Table II.** Summary of GPs’ key concerns and associated recommendations.

Concern 1: The impact of public health campaigns on primary care workload	GPs felt that campaigns would increase their workload and were concerned that many of the additional patient consultations would be inappropriate. To manage the extra workload, GPs felt that some financial resources invested in campaigns should be directed towards primary care to meet the extra demand for services
Concern 2: The impact on care pathways	GPs were concerned that secondary care services may become overwhelmed by extra referrals and that additional resources should be in place to deal with the additional workload
Concern 3: Causing undue anxiety amongst the public	GPs were concerned that members of the public may become unduly anxious about musculoskeletal problems. This may compel some people to seek help for conditions for which self-management would be more appropriate. GPs felt that this could be minimised by a campaign focused on those symptoms which were very specific for RA
Concern 4: Lack of knowledge about the early symptoms of RA	GPs felt that little was known about the symptoms that characterise the earliest stages of RA and suggested that research was needed to understand the way that symptoms emerge. They suggested that this information could be used to inform the content of public health campaigns and assist GPs in identifying those in need of a referral to a rheumatologist

### Theme 1. The need for early intervention and for a campaign

GPs recognised the need for early intervention in RA and that early diagnosis and referral of patients suspected of having RA had a number of long-term benefits:

“It is incumbent on us to ensure that for those where we are suspicious that they have access to specialists and treatments in a timely fashion, because it affects the prognosis, the course of their disease, and ultimately because of the nature of the disease their quality of life.”

GPs felt very strongly about the importance of early intervention for people with RA and recognised their role in the patient pathway. In addition, GPs recognised that there was a lack of awareness about RA amongst the general public, and that this was a barrier to early consultation.

“Finding a dodgy lump is a bad thing. People don't appreciate that having a hot painful joint, or several hot painful joints, can be a bad thing.”

However, despite GPs acknowledging the importance of early referral and treatment and the lack of public awareness about RA, GPs questioned the strength of the evidence regarding the impact of patients delaying in seeking help. GPs also questioned whether patients not presenting with the early symptoms of RA was the primary reason for patients not being started on treatment early. GPs particularly wanted data that patients were coming to harm because they had delayed in seeking help at RA onset.

“ I would only really be persuaded that some sort of education campaign were necessary if there was audit data demonstrating that a significant minority of people were coming to harm as a result of lack of awareness.”

GPs were keen to distinguish between two types of behaviour. The first, described as waiting for a period of time during which initially non-specific symptoms would evolve into something more worrisome, was viewed as a sensible approach. The second was described as a more neglectful behaviour with patients ignoring severe symptoms for a longer period of time.

“Because diseases emerge and evolve, and at the first presentation of the symptoms…. There is a point where the condition develops. I think there is a difference with delay and neglect … and there is another type of delay which is not necessarily neglecting the symptoms and ignoring the symptoms and deliberately not seeking care.”

### Theme 2: Concerns about a future help-seeking campaign for RA

Despite GPs recognizing the importance of early intervention, and the need for greater public awareness regarding RA, GPs had a number of concerns about a campaign to promote rapid help seeking for symptoms of RA. Theme 2 outlines four such areas of concern.

*Concern 1: A paucity of knowledge about the early symptoms of RA.* The concern most commonly discussed by GPs related to their understanding of the early symptoms of RA and the way that patients presented in the earliest phases of the disease. GPs recognised that they found the early symptoms of RA difficult to identify.

“I'm a bit conflicted because I don't know what a very early presentation looks like. I mean you get taught what it looks like but you think you only ever see that in patients who've had it for years.”

GPs desired more information on early symptoms, both to create a robust message for a help-seeking campaign about early RA, and also to enable them to distinguish more accurately between cases of suspected RA and other common conditions.

“In the end, get back to the patients and say well this is how it's emerging, these are the ways and the amber signs in terms of things to look for. Because one of the questions may be that over a four-week period or six-week period you may get a collection of these symptoms emerging. That's the point at which you need to do something.”

GPs’ perspectives were that the symptoms of early RA are often non-specific, making an early diagnosis difficult.

“In general practice a lot of what we deal with is undifferentiated presentation, uncertainty and so there will be some people who we have to manage over a period of time and it's about keeping that period of time short enough that it doesn't upset their prognosis but long enough that it allows the situation to become clear.”

GPs described the need to identify, for them, “the amber signs” of the onset of RA.

“What are the amber signs and the usual constellation of symptoms that you should look for? You almost have this sort of arthralgia plus ... what are the five things to look out for in the presentation of RA?”

*Concern 2: The impact of a campaign on primary care workload.* GPs recognised that strategies to increase awareness could be beneficial. However, they also highlighted that a campaign to promote help-seeking behaviour would increase their workload – particularly in the case of a campaign encouraging patients with common symptoms (e.g. joint pain and stiffness) to seek help.

“So the strengths are your mass coverage and your increased awareness by the population will undoubtedly mean that a couple of people will get through a door faster. The weaknesses are of course an increased workload in primary care because everybody who has done a bit of gardening who has got an achy joint will immediately think ‘aahhh’.”

In a resource-limited environment, GPs highlighted the cost associated with seeing more patients with musculoskeletal symptoms and that other aspects of their service were likely to suffer unless additional resources were available.

“There does need to be recognition that all the systems are by very nature resource limited and if you start pulling in lots of folks with symptoms for one specific area there will be an opportune cost, it's inevitable.”

*Concern 3: The impact on resources in steps along the patient care pathway distal to general practice.* It was highlighted that an increased volume of work in primary care would be likely to translate into an increased referral rate to secondary care. If secondary care services were not adequately resourced to meet this demand it was suggested that a bottleneck would build up that would be detrimental to patient care.

“They put these public health campaigns out without any thought about how it's going to be dealt with. They come to see the GP and I think all right, great, I'll refer them on. And then you think great there is no service, or no funding for the service.”

In particular, it was felt that if a help-seeking intervention had a low specificity for early RA, it may actually lead to people with genuine RA being seen later.

“If they find they can't get an appointment for six weeks because everybody that has got some minor joint pain has jumped in the queue first then it's not necessarily going to be that helpful for them. It would need to be thought through and evidence based.”

*Concern 4: Causing undue anxiety and inappropriate help seeking in those without early RA.* GPs were concerned about campaigns encouraging people not needing medical intervention (e.g. those whose symptoms would be naturally self-limiting) to seek help.

“If not well considered could draw in lots of people who actually either wouldn't have come to see us and potentially didn't need to and their symptoms would have played out over time in a different way. Because the flip side is that you are potentially exposing them to lots of investigations and health service contact they perhaps don't need.”

GPs were concerned that public health messages that caused people to question their health and look for suspicious symptoms could create unnecessary fear and anxiety.

“There is a danger with making people aware that people become fearful.”

“Usually you will attract the worried well, the highly educated and they just worry about everything and anything and the people you really want to attract you won't reach.”

GPs recognised that even a well-designed campaign would result in unnecessary consultations. However, they suggested that the success of a campaign could be judged by the proportion of those with early RA consulting in relation to the overall increase in workload.

“I mean the problem with any campaign is that you don't stop other people from coming who would have come anyway; all you do is just increase the number of people who come.”

### Theme 3: GPs’ perspectives on the features of “good” and “bad” campaigns

GPs valued campaigns that drew on very specific indicators of the illness in question.

“I think the FAST campaign [promoting rapid help-seeking at the first symptoms of stroke, i.e. Face, Arms, Speech and Time] to act fast if somebody's got symptoms of a stroke is actually extremely good because those are incredibly strong signs that they are promoting.”

While GPs emphasised that a strong evidence base to campaigns was necessary, they also valued simple, concise, and memorable messages.

“There has recently been advertising about CPR in the community, ‘Forget the kiss of life, just get on with the chest compressions’. It was brilliant because it was witty and it caught your attention because it was different. It caused a bit of controversy but it was extremely effective and it was based on an evidence base.”

Some GPs discussed the S factor campaign (also referred to as the 3S campaign). This campaign, developed by the Rheumatology Futures Research Group, used posters (primarily displayed in GP surgeries and rheumatology departments) that highlighted common symptoms of RA (joint Swelling, Stiffness and pain on Squeezing) (23). GPs felt this campaign could be improved by considering factors beyond the basic description of symptoms given. As outlined in theme 2, GPs were keen for a good campaign to specify the nature of the symptoms experienced, including their typical location and intensity.

“So what are for RA, what are the herald symptoms for RA, what are the early symptoms that you want patients to come to you to with…. But with the 3S campaign, they suggested what the symptoms were but did not suggest a threshold. It's not just about joint pain, it's about pain at specific joints like in the hands and feet.”

In addition, GPs highlighted campaigns that they considered to be poorly constructed, and had the potential to negatively impact on patients and on primary care services. GPs were concerned about the public being told that common non-specific symptoms indicated a need for a rapid GP consultation.

“I saw a ridiculous campaign about if you'd had a cough for three weeks or more see your GP as you need a chest X-ray…. From my point of view it's an absolutely ridiculous statement because what they don't say is that if you have had a cough for three weeks without a reason that you could explain. It didn't explain that if you've had a cough because you've had a stinking cold, a cough with hay fever I don't want to see you. I want to see you if you've got a new cough without any other factors.”

## Discussion

The evidence base for early DMARD initiation for RA is clear, and GPs largely accepted the rationale for developing a public health campaign to encourage people with RA to seek help rapidly. However, strong concerns were expressed about the lack of understanding of the earliest symptoms of RA and unduly worrying the public with poorly constructed media campaigns. Furthermore, GPs stressed that the broader impact of a campaign on rheumatological services in secondary care, and on the totality of the workload of GPs, needed to be considered. GPs believed that some previous campaigns had failed to address these and consequently had negatively impacted patients.

To date, no research has been carried out to explore the concerns that GPs have about campaigns to promote help-seeking behaviour at the onset of chronic conditions such as RA. The literature surrounding campaigns is weighted towards the reporting of positive effects such as the number of new cases identified (27). This is surprising as health campaigns have a direct impact on primary healthcare services, and, in many countries, GPs are the gate-keepers to specialist services. For example, according to the Commonwealth Fund's report on international profiles of health care systems, countries such as Demark, the UK, and Australia have GPs as gate-keepers to specialist services, whereas in countries such as Germany and Sweden gate-keeping services are optional but incentivised (28,29). Therefore, whilst the findings of this study may be applicable to many healthcare settings, further exploration is needed of health professionals’ views on campaigns to promote help-seeking behaviour in countries where direct access to specialist services are the norm.

There are clear barriers to launching media-based health campaigns. The extra resources needed in primary care and in secondary care is one of the more obvious. In addition, this study also highlights that GPs feel that they need more information about the symptom clusters and the positive and negative predictive values of common RA symptoms in the community. Many qualitative studies have observed that the symptoms at the onset of RA are varied and often non-specific (30). Indeed, a recent report from the EULAR Study Group on Risk Factors for RA has highlighted the need for additional research in this area (31) This is vital as it is estimated that 15% of GP consultations are for musculoskeletal problems (32), yet, given the incidence of RA (40 per 10 000 per year) (33), a typical full-time GP in the UK will see approximately one new RA patient per year (34,35). A qualitative exploration of the reasons why primary health providers in the US were reluctant to refer to secondary care included their uncertainty about the clinical characteristics of early RA, particularly when symptoms were mild and only slowly progressive (36). Difficulties in identifying the clinical characteristics of early RA may pose a difficulty for the messages contained in an intervention to promote help-seeking behaviour.

This qualitative study identified a broad range of concerns that GPs have about public health intervention for RA. There are limitations to the generalizability of these findings, therefore the themes identified here should be used to inform quantitative explorations of GPs’ knowledge of RA (particularly their views on the early symptoms of RA), their views on patient delay in their locality, their perceptions about barriers to early referral to secondary care, and their thoughts on campaigns to promote early presentation giving special consideration to local needs, and the capacity and structure of local health services. Importantly this study highlights the value of gaining GPs’ perspectives when considering the development and launch of help-seeking campaigns – perspectives that have been poorly captured in the literature to date.
